# The Peptide/Antibody-Based Surface Decoration of Calcium Phosphate Nanoparticles Carrying siRNA Influences the p65 NF-κB Protein Expression in Inflamed Cells In Vitro

**DOI:** 10.3390/biomedicines10071571

**Published:** 2022-07-01

**Authors:** Elena K. Müller, Nataniel Białas, Matthias Epple, Ingrid Hilger

**Affiliations:** 1 Department of Experimental Radiology, Institute of Diagnostic and Interventional Radiology, Jena University Hospital, Friedrich Schiller University Jena, Am Klinikum 1, D-07740 Jena, Germany; elena-mueller@posteo.de; 2 Inorganic Chemistry and Center for Nanointegration Duisburg-Essen (CENIDE), University of Duisburg-Essen, Universitaetsstrasse 5-7, D-45117 Essen, Germany; nataniel.bialas@uni-due.de

**Keywords:** RGDs, CD69, IgGs, integrins, p65 NF-κB, gene silencing, inflammation, calcium phosphate, silica, nanoparticles, siRNAs

## Abstract

Earlier studies with nanoparticles carrying siRNA were restricted to investigating the inhibition of target-specific protein expression, while almost ignoring effects related to the nanoparticle composition. Here, we demonstrate how the design and surface decoration of nanoparticles impact the p65 nuclear factor-kappa B (NF-κB) protein expression in inflamed leucocytes and endothelial cells in vitro. We prepared silica-coated calcium phosphate nanoparticles carrying encapsulated siRNA against p65 NF-κB and surface-decorated with peptides or antibodies. We show that RGD-decorated nanoparticles are efficient in down-regulating p65 NF-κB protein expression in endothelial cells as a result of an enhanced specific cellular binding and subsequent uptake of nanoparticles. In contrast, nanoparticles decorated with IgG (whether specific or not for CD69) are efficient in down-regulating p65 NF-κB protein expression in T-cells, but not in B-cells. Thus, an optimized nanoparticle decoration with xenogenic IgG may stimulate a specific cellular uptake. In summary, the composition of siRNA-loaded calcium phosphate nanoparticles can either weaken or stimulate p65 NF-κB protein expression in targeted inflamed leucocytes and endothelial cells. In general, unveiling such interactions may be very useful for the future design of anti-p65 siRNA-based nanomedicines for treatment of inflammation-associated diseases.

## 1. Introduction

It is generally known that the immune system is a complex network of diverse cell types, signaling pathways, and effector molecules, which are all necessary to provide a defense against foreign pathogens. Important cellular players are endothelial cells, phagocytic cells, such as monocytes, macrophages, dendritic cells, etc., which are able to recognize and respond to a multitude of antigens (innate immunity), as well as B- and T- lymphocytes, plasma cells, etc., which are part of adaptive immunity [1].

In particular, the nuclear factor-kappa B (NF-κB) is known to play a crucial role during immune and inflammatory responses, cell growth, survival and development [2]. Herein, NF-κB particularly regulates the expression of pro-inflammatory cytokines, matrix-degrading enzymes (matrix metalloproteinases; MMPs), adhesion molecules, and further mediators, which all determine the initiation and perpetuation of chronic inflammation [3]. For such reasons, a specific NF-κB blockade is considered to be of particular importance for therapeutic interventions in inflammatory diseases [4,5]. NF-κB plays a critical role in development, survival, differentiation, and activation of B lymphocytes as well as T lymphocytes [6,7,8]. In endothelial cells, the NF-κB pathway is essential for the response in stress situations in the initial stages of inflammatory and coagulatory processes [9]. Among the different members of the NF-κB pathways, p65 is known to be a critical player [10]. As an intracellular protein, it is quite challenging to therapeutically address p65. For this reason, the application of small interfering RNA (siRNA) has been suggested with the aim to specifically degrade p65 mRNA after transcription by RNA interference (RNAi) and prevent its translation thereafter [11]. In order to protect it from degradation in the blood, siRNA can be encapsulated into calcium phosphate nanoparticles with their inherent capacity to transport it into cells [12]. 

Owing to the fact that NF-κB is a pleiotropic mediator [13,14], siRNA-carrying nanoparticles should be directed specifically to the immune cells of interest, e.g., by the aid of dedicated surface decorations. Such decorations encompass dedicated ligands for receptor molecules on the target cells. Of these, integrins are good vascular targeting moieties, because they are specifically overexpressed in endothelial cells during inflammation. It has been shown that peptides containing the RGD (Arg-Gly-Asp) sequence interact, with especially high affinity, with integrin receptors [15] and that they recognize integrin heterodimers between the αV unit (CD51) and the β3 unit (CD61) on the endothelial cell surface, which is a receptor for vitronectin and also involved in angiogenesis [16]. Moreover, CD69 is a classical early marker of lymphocyte activation due to its rapid appearance on the surface of the plasma membrane after stimulation. It is a membrane-bound type II C-lectin receptor, which is expressed by several subsets of tissue-resident immune cells, such as different T-cell subsets, determining the migration-retention ratio as well as the acquisition of effector or regulatory phenotypes [17]. In addition to mature T cells, CD69 is inducibly expressed by immature T cells, B cells, natural killer (NK) cells, monocytes, neutrophils, and eosinophils, and also constitutively expressed by a subset of resident T cells and platelets [18,19].

Earlier studies with nanoparticles carrying siRNA were focused on the feasibility to inhibit the target protein expression, while almost ignoring the nanoparticles composition. In the present study, we investigated how the composition of the targeting moiety (e.g., RGD, CD69, IgG; either a peptide or an antibody) on the surface of calcium phosphate nanoparticles impacts the p65 NF-κB protein expression in inflamed leucocytes and endothelial cells, such as B-cells, endothelial cells, and T-cells in vitro, and how such effects can either increase or even weaken the p65-specific functionality of the encapsulated functional siRNA. It is of uttermost importance to reveal such interactions to optimize nature and surface functionalities of therapeutic nanoparticles when addressing different immune cells with NF-κB-specific siRNAs.

## 2. Materials and Methods

### 2.1. Reagents for Nanoparticle Synthesis

Ultrapure sterile nuclease-free water (Invitrogen, Waltham, MA, USA) was used for all syntheses unless otherwise noted. All syntheses and handling of nanoparticles were performed in a nuclease-free environment (RNase AWAY; Carl Roth, Karlsruhe, Germany). All glassware used for synthesis and handling of the nanoparticles was heat-sterilized and depyrogenated (250 °C, 1 h) before use. The following chemicals were used for synthesis and preparation of bioactive calcium phosphate nanoparticles: calcium lactate pentahydrate (Sigma-Aldrich, Burlington, MA, USA), diammonium hydrogen phosphate (VWR, Radnor, PA, USA), branched polyethyleneimine (PEI; *M*_w_ 25 kDa; Sigma-Aldrich, Burlington, MA, USA), Cy5-labeled branched PEI (*M*_w_ 25 kDa; Surflay Nanotec, Berlin, Germany), tetraethoxysilane (TEOS, Sigma-Aldrich, Burlington, MA, USA), ethanol (99%; Fisher Chemicals, Waltham, MA, USA), aqueous ammonia solution (30–33%; Carl Roth, Germany), (3-mercaptopropyl)trimethoxysilane (MPS; Sigma-Aldrich, USA), sulfo-N-succinimidyl 4-(maleimidomethyl)cyclohexane-1-carboxylate sodium salt (sulfo-SMCC; Iris Biotech GmbH, Germany), and D-(+)-trehalose dihydrate (VWR, USA). For nanoparticle analysis by atomic absorption spectroscopy (AAS), the particles were dissolved in a 3:1 *v*:*v* mixture of H_2_O:HCl (37%; VWR, USA). For dynamic light scattering (DLS) and ζ-potential analyses, aqueous dispersions of the nanoparticles were used. Scanning electron microscopy (SEM) imaging was done on dried nanoparticles. UV/Vis spectrophotometric analyses were performed with supernatants obtained after nanoparticle centrifugation and ultracentrifugation. Functional siRNA against p65 NF-κB was obtained as functional and control (scrambled) silencing ribonucleic acid from Santa Cruz Biotechnology (USA). Functional siRNA (sc29411; *M*_w_ 13.8 kDa; denoted as siRNAf in the following) was a mixture of 4 target-specific siRNA duplexes with the following sequences (from 5′→3′): CCAUGGAGUUCCAGUACUUtt, UCAGCACCAUCAACUUUGAtt, CGAAGUGCGUACACAUUCUtt, GGAUUCCUGUACACCUUGAtt. A non-targeting 20–25 nt siRNA designed as negative control with proprietary base sequence (sc 37007; denoted as siRNAs in the following) was also obtained from Santa Cruz Biotechnology. For nanoparticle surface decoration, either cRGDfK-peptide [cyclo(-Arg-Gly-Asp-D-Phe-Lys)] (*M*_w_ 603 Da; BACHEM AG, Bubendorf, Switzerland), IgG monoclonal anti-CD69, its ĸ isotype control antibodies (both from Armenian hamster, BioLegend®, San Diego, CA, USA; *M*_w_ 150 kDa), or a murine IgG-ĸ-IC (Bioscience, San Diego, CA, USA; *M*_w_ 150 kDa) were used.

### 2.2. Synthesis of Ligand-Decorated Calcium Phosphate Nanoparticles for Gene Silencing

**Figure 1:**
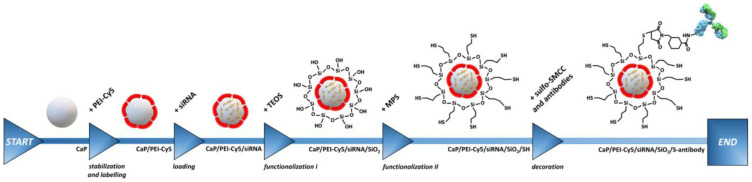
Schematic representation of the synthesis of fluorescent ligand-decorated and siRNA-loaded calcium phosphate nanoparticles (CaP/PEI-Cy5/siRNA/SiO_2_/S-peptide/antibody) for in vitro cell targeting and gene silencing of p65 NF-κB in murine cells. CaP—calcium phosphate, Cy5—cyanine 5, MPS—(3-mercaptopropyl)trimethoxysilane, PEI—polyethyleneimine, siRNA—small interfering RNA, sulfo-SMCC—sulfo-N-succinimidyl 4-(maleimidomethyl) cyclohexane-1-carboxylate sodium salt, TEOS—tetraethoxysilane. The image of the antibody was adapted from static.turbosquid.com.

The synthesis of bioactive silica-coated calcium phosphate nanoparticles was performed by wet-chemical precipitation as described in detail in Refs. [12,20]. Briefly, polyethyleneimine (PEI)-coated calcium phosphate nanoparticles CaP/PEI-Cy5 were prepared from aqueous solutions. These nanoparticles were fluorescent as we used Cy5-functionalized polyethyleneimine (PEI-Cy5). Next, these cationic particles were loaded with the corresponding siRNA (negatively charged) that adsorbed on the particle surface. These CaP/PEI-Cy5/siRNA nanoparticles were subsequently coated with a silica layer by a modified Stöber synthesis with TEOS to protect the siRNA from enzymatic degradation. The resulting CaP/PEI-Cy5/siRNA/SiO_2_ nanoparticles were covalently functionalized by silanization with thiol groups (-SH) to enable surface decoration of the nanoparticles with ligands, i.e., peptides or antibodies (Figure 1). In brief, 1 mL of CaP/PEI-Cy5/siRNA/SiO_2_ or CaP/PEI-Cy5/SiO_2_ nanoparticle dispersion was added to a stirred mixture of 4 mL absolute ethanol and 5 μL MPS (conjugation reagent) and further stirred overnight at room temperature (RT) in darkness. After this time, the nanoparticles were collected by centrifugation (1537× *g*, 30 min, RT), and the nanoparticle pellet was re-dispersed in 0.5 mL water, followed by vortexing and ultrasonication (cycle 0.8, amplitude 70%, 4 s). Prior to coupling with the nanoparticles, ligands (peptides/antibodies) were first activated in a reaction with the heterobifunctional crosslinker sulfo-SMCC, which contains an N-hydroxysuccinimide (NHS) ester and a maleimide functional group. This enables a covalent conjugation between amine-containing ligands and thiol-functionalized nanoparticles. In total, 0.25 mL ligand solution (0.5 mg/mL) was mixed with 0.125 mL sulfo-SMCC (4 mM) and left for activation for 4 h (RT, no stirring). Next, the activated ligand was purified by spin filtration (Amicon^®^ Ultra 0.5 mL; regenerated cellulose 3000 NMWL; Merck Millipore Ltd., Dublin, Ireland) to remove free sulfo-SMCC. The spin filter was first activated (14,064× *g*) with water. Then, the activated complex was spin-filtered by centrifugation (14,064× *g*), washed with 0.4 mL water and centrifuged to remove residual sulfo-SMCC. Finally, the spin filter was turned upside down, placed in a new tube, and the activated ligand was detached by centrifugation (983× *g*, 2 min, 4 °C). Before the activated ligand was reacted with the thiol-functionalized nanoparticles, it was analyzed by UV spectroscopy (NanoDrop) at *λ*_max_ = 205 nm (E0.1%) and 280 nm (E1%) to determine the ligand concentration. In total, 0.5 mL of thiol-functionalized nanoparticles was mixed with 0.25 mL activated ligand solution and incubated for 24 h at 4 °C in darkness. The surface-functionalized nanoparticles were collected by centrifugation (21,041× *g*, 30 min, 4 °C), followed by re-dispersion in 0.5 mL water, followed by vortexing and gentle ultrasonication. The residual supernatant ultracentrifugation was analyzed for the presence of the free (unbound) ligand by UV spectroscopy (NanoDrop) to determine the concentration of nanoparticle-conjugated ligands. The supernatants were analyzed for the presence of free siRNA to determine the siRNA concentration in the nanoparticles during this multi-step synthesis. Finally, the nanoparticles were aliquoted, freeze-dried, and stored at −80 °C until application.

A thorough analysis of the calcium phosphate nanoparticles indicated a loss of calcium during the washing steps (purification) and their re-dispersion during synthesis and processing for their further chemical modification (silica-functionalization, thiol-functionalization, and ligand-decoration steps). Neither the silica shell nor a consistent pH adjustment to 10 prevented the calcium loss, which was significant after purification of silica-coated nanoparticles (up to 71% of calcium was lost during these procedures). Moreover, working at higher pH of 10 did not prevent the calcium loss. A thorough step-by-step investigation of this effect revealed the nature of the calcium loss, which varied between 25–60% per synthesis step (Supplementary Figure S1). After several modifications of the synthetic steps, we excluded all purification steps by washing and increased the concentration by a factor of 2 compared to the earlier synthesis [12]. This increased the content of calcium in the final nanoparticle sample by a factor of 2 to 3. Note that all synthetic steps were performed in small scale due to the low amount of costly siRNA.

### 2.3. Nanoparticle Characterization

The nanoparticle characterization was performed by DLS and zeta potential (ζ) determination in order to assess the nanoparticle size and colloidal stability (Zetasizer Nano ZS; Malvern Panalytical, Germany; laser wavelength *λ* = 633 nm; Smoluchowski approximation; refraction index of hydroxyapatite *n* = 1.65, absorption 0.01). SEM imaging was performed with an ESEM Quanta 400 FEG microscope (FEI, Hillsboro, OR, USA) on gold/palladium (80:20)-sputtered samples at an accelerating voltage of 30 kV. Calcium (Ca^2+^) was determined by AAS with an iCE 3000 M-Series spectrometer (Thermo Scientific, Waltham, MA, USA). The efficiencies of nanoparticle loading with PEI-Cy5 and siRNA and the nanoparticle decoration with ligands were determined by UV/Vis spectrophotometry with a DS-11 FX+ device (DeNovix^®^, Wilmington, DE, USA). The endotoxin concentration in the nanoparticles was determined with an Endosafe^®^ Nexgen-PTS™ spectrophotometer (Charles River, Waltham, MA, USA), based on the limulus amoebocyte lysate (LAL) chromogenic assay. For a 20 g mouse, 0.1 EU (endotoxin units) is the maximum endotoxin level considered as safe [21], and we took care that our particles were always below this threshold. Reglo peristaltic pumps (Ismatec, Germany) were used for dosing the reagent solutions during synthesis. Centrifugation of the nanoparticles was carried out with a Rotofix 32A centrifuge (Andreas Hettich GmbH, Tuttlingen, Germany) and a Heraeus Fresco 21 ultracentrifuge (Thermo Scientific, USA), respectively. Nanoparticle pellets were re-dispersed with an UP50H ultrasonic processor (sonotrode MS1; Hielscher Ultrasonics GmbH, Teltow, Germany). Freeze-drying of the nanoparticles was carried out with a Christ Alpha 2–4 LSC instrument (Martin Christ GmbH, Osterode am Harz, Germany). Lyophilized nanoparticles were stored at −80 °C before application. Nanoparticles were lyophilized with D-(+)-trehalose as cryoprotectant. Immediately before application, the nanoparticles were re-dispersed in the same volume of water as present before freeze-drying under thorough vortexing. To calculate the concentration of nanoparticles, the Ca^2+^ concentration was measured by AAS and then numerically converted to the most common calcium phosphate phase hydroxyapatite, Ca_10_(PO_4_)_6_(OH)_2_ (see [12] for details).

### 2.4. Cell Lines and Culture Conditions

The murine monocyte cell line J774A.1 (CLS Cell Lines Service GmbH, Eppelheim, Germany) was cultured in DMEM/F-12 (1 + 1) (+)-L-glutamine (Gibco, Germany) supplemented with 10 vol% fetal calf serum (FCS; Gibco). The murine endothelial cell line SVEC4-10 (American Type Culture Collection, ATCC, 141 CCL81, Rockville, MD) was cultured in DMEM (1x) + GlutaMAX^TM^-I (Gibco, Germany) supplemented with 10 vol% heat-inactivated FCS. The murine T-lymphocyte (lymphoma) cell line TK-1 (ATCC) was cultured in RPMI 1640 medium supplemented with 10 vol% FCS, 0.1 mM non-essential amino acids, and 0.05 mM 2-mercaptoethanol (Thermo Fisher, Germany). The murine B lymphocyte (myeloma) cell line MOPC-315 (ATCC) was cultured in DMEM (Gibco, Germany) supplemented with 10 vol% FCS. All cells were cultured at 37 °C in 5% CO_2_ atmosphere.

### 2.5. Nanoparticle Uptake and Cell Viability

Cells were seeded in 24-well cell culture plates and pre-incubated for 24 h. Fluorescently labeled nanoparticles were added at 0.125–1.0 mg/L Ca^2+^. After a further 24 h of incubation, the cells were enzymatically harvested, washed with PBS, and stained with annexin-V-FLUOS (Roche Diagnostics, Mannheim, Germany) according to manufacturer’s recommendations for detection of dead (apoptotic) cells. Cellular nanoparticle uptake and cytotoxicity were analyzed by flow cytometry (Accuri C6 flow cytometer; BD Biosciences, Franklin Lakes, NJ, USA).

### 2.6. Incubation of Cells with siRNA-Loaded Nanoparticles

To study the impact of nanoparticles carrying functional p65 siRNA on the different cell types, the following experimental routines were followed: Prior to the investigations, cells were stimulated to induce an inflammation status: (I) SVEC4-10 cells were incubated with 10 µg/mL lipopolysaccharide (LPS) for 4 h; (II) TK-1 cells were stimulated with 5 µg/mL CD3ε-Biotin antibody and 2 µg/ mL CD28 antibody for 6 h prior to investigation, (III) MOPC-315 cells were incubated with 0.5 µM ODN-2006 and 75 ng/mL IL-4 for 3 h. Stimulated cells were subsequently exposed either to: (I) the decorated nanoparticles carrying functional siRNA (siRNAf, final concentration: 1 µg/mL, 72 h) to assess their impact on p65 protein expression; (II) decorated nanoparticles carrying non-functional, scrambled siRNA (siRNAs, final concentration: 1 µg/mL, 72 h) to control p65-specific functionality of encapsulated siRNAf; III) decorated nanoparticles containing no siRNA to control their impact of the unloaded nanoparticles on the p65 protein expression; (IV) SH-functionalized nanoparticles containing no siRNA, to control the effect of the free antibody coupling linker on target T-cells; (V) functional or non-functional p65 siRNA dissolved in the transfection agent Lipofectamine^TM^ (1 µg/mL, Thermo Fisher, Germany) to control the impact of free (non-encapsulated) siRNA with good availability in the cytoplasm; and (VI) Lipofectamine^TM^ (1 µg/ mL) without siRNA to control the impact of this substance per se on p65 protein expression. The nanoparticle concentration in the experimental arms (I) to (IV) was estimated by the Ca^2+^ component concentration, the corresponding values were 0.8–1.0 mg/L Ca^2+^. After nanoparticle treatment, the cells were lysed for protein isolation with RIPA buffer. The total protein concentration was measured with the Bradford assay. Cell lysates were used to measure protein levels.

### 2.7. p65 Protein Expression after Incubation with siRNA-Loaded Nanoparticles

After treatment, the cells were lysed for protein isolation with a peqGOLD TriFastTM reagent (VWR, Germany). The total protein concentration was measured via the Bradford assay. After electrophoretic separation (10% (*w*/*v*) SDS-Page, 20 µg total protein per lane) and Western blotting, proteins were probed with antibodies against p65 NF-κB (Santa Cruz Biotechnology, Heidelberg, Germany) and β-actin (Abcam, Cambridge, UK). The p65 expression of the different treatment groups was analyzed densitometrically by the ImageJ-software (NIH, Bethesda, MD, USA). Furthermore, the regulation of protein expression (up- or down-regulation) was calculated as the ratio of the p65 protein expression with respect to the non-treated inflammatory condition set to zero.

### 2.8. Competition Experiments

SVEC4-10 cells (3.5·10^5^ per mL) were incubated with cRGD-decorated nanoparticles (0.25 or 0.5 µg/mL RGD-peptide) and a 100-fold excess of free RGD-peptide (Bachem, Switzerland). TK-1 and MOPC-315 cells (4.0·10^5^ per mL) were incubated with nanoparticles decorated with CD69-IgG (from Armenian hamster) or IgG (human, 0.5 or 1.8 µL/mL IgG) and a 100-fold excess of free IgG (BioLegend^®^, USA). Incubation was carried out for 3 h at 37 °C, together with vortexing the cell suspensions every 30 min. Cellular nanoparticle uptake was measured by flow cytometry as described above.

### 2.9. Expression of Affinity Molecules on the Surface of Target T-Cells

The expression of integrin αVβ3 on SVEC4-10 cells and CD69 on MOPC-315 and TK-1 cells was investigated as control for the presence of the specific anchor of our decorated nanoparticles on the cell surface. For this, SVEC4-10 cells were enzymatically harvested and stained with an anti-mouse CD51 antibody (from rat, PE-labeled, 0.01 µg/µL, BioLegend^®^, USA) or the IgG isotype (isotype control, IgG1, κ Isotype, from rat, PE-labeled, 0.01 µg/µL, BioLegend^®^, USA) on ice in the dark for 30 min. MOPC-315 and TK-1 were harvested, centrifuged (200× *g*, 5 min, 4 °C), and washed with 1 (*w*/*v*) % BSA in PBS. Cells were stained with anti-mouse CD69 (from Armenian hamster, PE-labeled, 2.5 ng/µL, BioLegend^®^, USA) or the IgG isotype (isotype control, from Armenian hamster, PE-labeled, 2.5 ng/µL, Biolegend, USA) for 30 min on ice in the dark. Expression of activation markers on the cell surface was analyzed by flow cytometry, as described above.

### 2.10. Statistics

Data requiring statistical analyses were evaluated with the Prism 9 program (GraphPad software, San Diego, CA, USA). All data were considered to be normally distributed based on literature reports on normality distribution of the same variables. A Student’s *t*-test or an ANOVA (analysis of variance) with Tukey’s post hoc test was used to compare groups. Differences with *p*-values of 0.05 or less were considered as statistically significant.

## 3. Results

### 3.1. Nanoparticles

**Figure 2:**
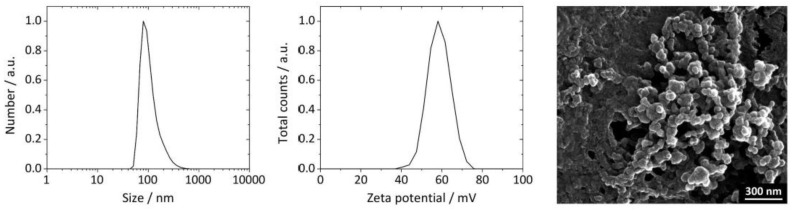
Physicochemical properties of representative ligand-decorated and siRNA-loaded calcium phosphate nanoparticles. Normalized particle size distribution (left) and the corresponding zeta potential (center) by DLS, and SEM micrograph of CaP/PEI-Cy5/siRNAs/SiO_2_/S-IgG-anti-CD69-CTRL nanoparticles (right). DLS and SEM results of all other types of nanoparticles investigated in this study are given in Supplementary Figures S2 and S3. For detailed nanoparticle characterization data, see Table 1 and Table 2.

The synthesis of bioactive silica-coated calcium phosphate nanoparticles was highly reproducible, as performed by wet-chemical precipitation (see Section 2 and Figure 1). In general, the nanoparticles were spherical (diameter 36 to 67 nm by SEM), monodisperse (PDI < 0.35), colloidally stable (zeta potential between +27 and +41 mV), and non-pyrogenic (<0.1 EU per mL) (Figure 2, Table 1 and Table 2, Supplementary Figures S2 and S3).

The efficiency of siRNA encapsulation into the nanoparticles was up to 90%. The efficiency of the nanoparticle surface decoration with RGD peptides (cyclic, cRGDfK, see Section 2) and with IgG antibodies was ∼95% and ∼50%, respectively.

### 3.2. The Impact of RGD-Peptide Decorated and Functional (p65 siRNAf) Nanoparticles on Endothelial Cells

**Figure 3:**
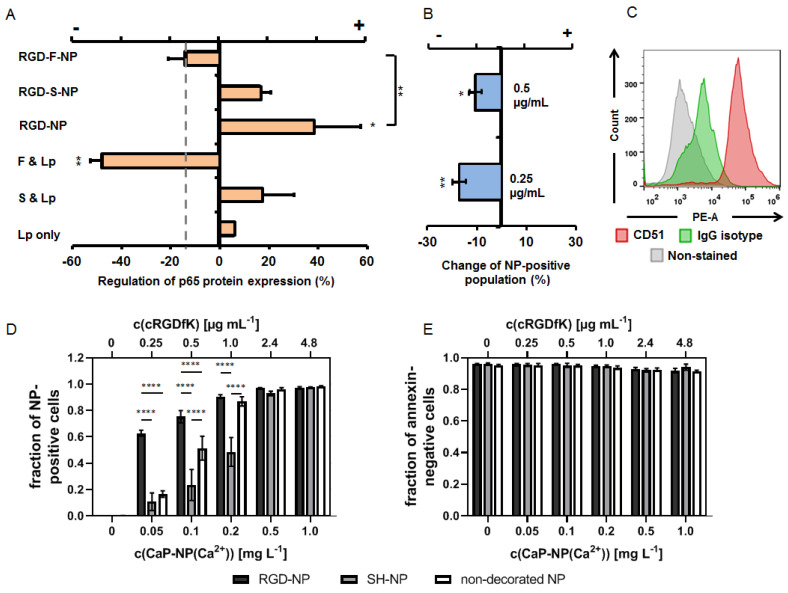
The potential of RGD-decorated CaP/PEI-Cy5/SiO_2_ nanoparticles to down-regulate NF-kappa B p65 protein expression in murine endothelial cells (SVEC4-10). (**A**) Potential of nanoparticles to down-regulate p65 together with each of their components as determined via immunoblotting, nanoparticle concentration: 1 µg/mL siRNA. For detailed specification of nanoparticle formulations used see Table 1. F = functional siRNA, S = scrambled siRNA, Lp = Lipofectamine^TM^. The down-regulation is the ratio of the p65 expression with respect to the inflammatory condition (LPS-primed cells, absence of nanoparticles) set to zero. “+ regulation” = up-regulation; “- regulation” = down-regulation. The dashed line depicts the non-inflammatory condition. (**B**) RGD-specific-binding of the decorated nanoparticles as determined by flow cytometry (change of nanoparticle-positive cells under competition condition (non-competition condition = 0)). (**C**) Control for the expression of CD51 (integrin αV) on the endothelial cell surface as target molecule of RGD-NP determined via flow cytometry. (**D**) Nanoparticle uptake. (**E**) Nanoparticle biocompatibility (annexin-negative cells). Statistical differences between control or the indicated groups with * *p* < 0.05, ** *p* < 0.01, **** *p* < 0.0001.

In endothelial cells, the RGD-decorated and functional (p65 siRNAf) nanoparticles down-regulated the p65 NF-κB protein expression with respect to non-nanoparticle exposed cells (LPS-primed cells only, set to zero), albeit to a lesser extent when cells were transfected with p65 functional siRNA in the presence of Lipofectamine^TM^ at equivalent concentrations (1 µg/mL, Figure 3A). The nanoparticle decoration with RGD per se stimulated p65 NF-κB expression, but the additional presence of non-functional siRNA in the nanoparticle configuration attenuated this effect. The nanoparticle binding was, at least in parts, specific for α5β3-integrin, as seen by competition assays and the analyses for the presence of integrin αV by flow cytometry (Figure 3B,C). The mentioned impact of the nanoparticles on the p65 NF-κB expression was the result of RGD-mediated binding and uptake by endothelial cells, since a larger number of nanoparticle-positive cells was observed compared to the exposure of cells to non-RGD-decorated nanoparticles (Figure 3D). Furthermore, a bias on the mentioned effects due to nanoparticle cytotoxicity can be excluded (Figure 3E).

### 3.3. The Impact of Antibody-Decorated and Functional (p65 siRNAf) Nanoparticles on T- and B-Cells

**Figure 4:**
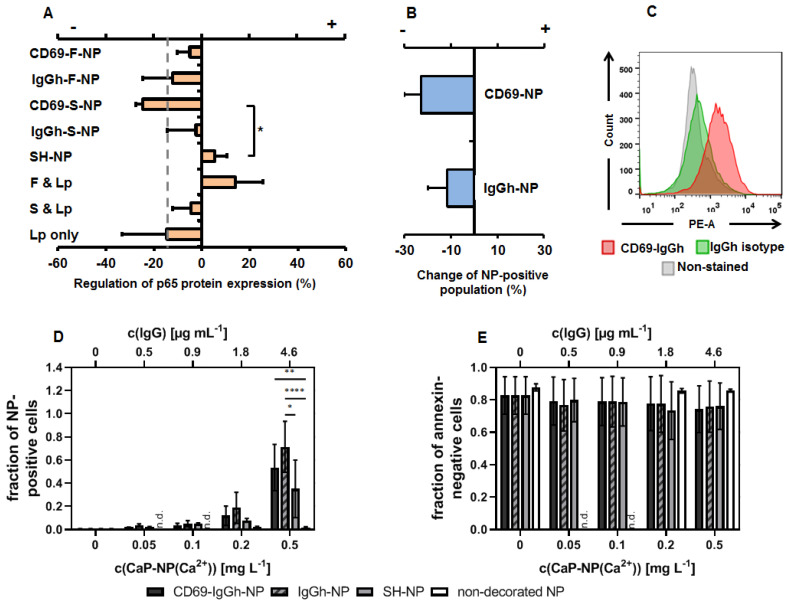
The potential of CD69-decorated CaP/PEI-Cy5/SiO_2_ nanoparticles to down-regulate NF-kappa B p65 protein expression in murine T-cells (TK-1). (**A**) Potential of nanoparticles to down-regulate p65 together with each of their components as determined via immunoblotting, nanoparticle concentration: 1 µg/mL siRNA. For detailed specification of nanoparticle formulations used see Table 1 and Table 2. F = functional siRNA, S = scrambled siRNA, Lp = Lipofectamine^TM^. The regulation is the ratio of the p65 expression with respect to the inflammatory condition (CD3/CD28-stimulated cells, absence of nanoparticles) set to zero. “+ regulation” = up-regulation; “- regulation” = down-regulation. The dashed line depicts the non-inflammatory condition. (**B**) Specificity of CD69 or FcγR-specific-binding of the decorated nanoparticles as determined via flow cytometry (change of nanoparticle-positive cells under competition condition (non-competition condition = 0)). (**C**) Control for the expression of CD69 on T-cells surfaces as target molecule of decorated nanoparticles determined via flow cytometry. (**D**) Nanoparticle uptake. (**E**) Nanoparticle biocompatibility (annexin-negative cells). Statistical differences between control or indicated groups with * *p* < 0.05, ** *p* < 0.01, **** *p* < 0.0001. n.d.: not determined.

In T-cells, all IgG-decorated nanoparticles resulted in a down-regulation of p65 NF-κB protein expression with respect to non-nanoparticle exposed cells (stimulated cells only, set to zero) independently of whether or not the siRNA was specific for p65 (siRNAf or siRNAs). Obviously, the combination of IgG and siRNA was favorable to down-regulate p65 NF-κB protein expression in those nanoparticle-exposed cells (Figure 4A). In contrast, p65 functional siRNA transfected with Lipofectamine^TM^ (Thermo Fisher Scientific) rather up-regulated p65 in T-cells, whereas non-functional siRNA did not. Moreover, the further nanoparticle controls had ambiguous effects on p65 NF-κB protein expression, depending on the fact of being native nanoparticles, free p65 functional siRNA transfected with Lipofectamine™ or Lipofectamine™ per se. The nanoparticle accumulation in T-cells was, at least in parts, specific for CD69 and for other antibody binding sites at the cell surface (e.g. FcγR), as detected via competition experiments (presence of excess of free IgG targeting or not CD69) together with the corroboration of the expression of CD69 on the T-cell surface via flow cytometry (Figure 4B,C). There was an increased accumulation of IgG-decorated nanoparticles in T-cells (with or without specificity for CD69) in comparison to native nanoparticles (SH or non-decorated, Figure 4D). Moreover, a clear concentration dependency up to 0.5 mg/L Ca^2+^ and a good biocompatibility (no cytotoxicity, Figure 4E) were found.

**Figure 5:**
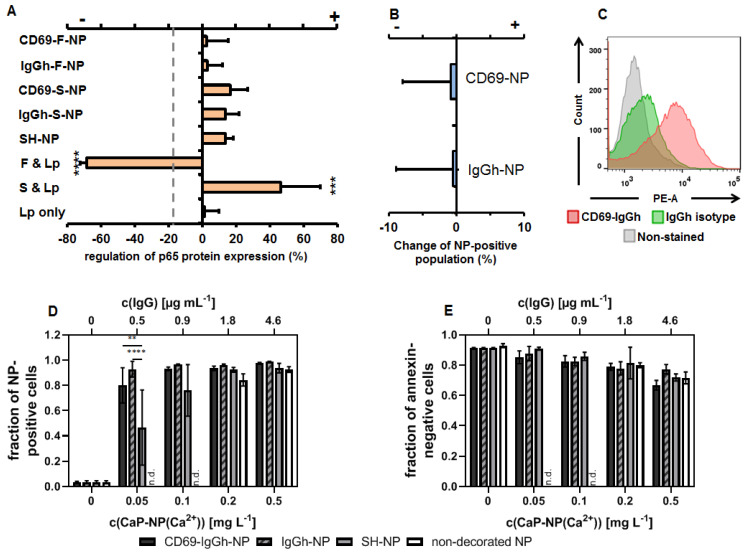
Uptake behavior of CD69-decorated CaP/PEI-Cy5/SiO_2_ nanoparticles and potential to down-regulate NF-kappa B p65 protein expression in murine B-cells (MOPC-315). (**A**) Potential of nanoparticles to down-regulate p65 together with each of their components as determined via immunoblotting, nanoparticle concentration: 1 µg/mL siRNA. For detailed specification of nanoparticles formulations used see Table 1 and Table 2. F = functional siRNA, S = scrambled siRNA, Lp = Lipofectamine^TM^. The regulation is the ratio of the p65 expression with respect to the inflammatory condition (ODN-2006/IL-4-stimulated cells, absence of nanoparticles) set to zero. “+ regulation” = up-regulation; “- regulation” = down-regulation. The dashed line depicts the non-inflammatory condition. (**B**) Specificity of CD69 or FcγR-specific-binding of the decorated nanoparticles as determined via flow cytometry (change of nanoparticle-positive cells under competition condition (non-competition condition = 0)). (**C**) Control for the expression of CD69 on B-cells surface as target molecule of decorated nanoparticles determined via flow cytometry. (**D**) Nanoparticle uptake. (**E**) Nanoparticle biocompatibility (annexin-negative cells). n.d.: not determined. Statistical differences between control or indicated groups with ** *p* < 0.01, *** *p* < 0.001, **** *p* < 0.0001.

In B-cells, the exposure to nanoparticles with 1 µg/mL p65 functional siRNA was not sufficient to down-regulate of the expression of p65 NF-κB. Instead, there was a slight up-regulation of p65, independent from the nanoparticle decoration with IgG (with or without CD69 antigen specificity), or the encapsulation of siRNA (p65 functional or non-functional) into them. The presence of native nanoparticles up-regulated the p65 protein expression above the level of non-nanoparticle-exposed but stimulated B-cells. Only the Lipofectamine^TM^-mediated transfection of p65 functional siRNA was very efficient in those cells (control experimental arm) (Figure 5A). Furthermore, there was no specificity in B-cell uptake of such decorated nanoparticles, neither with CD69 nor with IgG-isotype-decoration, although the investigated B-cells expressed CD69, as shown via flow cytometry (Figure 5B,C). Beyond this, the nanoparticle decoration with IgG (whether specific or not for CD69) generally increased their binding and uptake in B-cells, and there was no concentration dependency of nanoparticle binding from 0.05 mg/L Ca^2+^ and above (Figure 5D). The nanoparticles showed a good biocompatibility and only a slight cytotoxic effect on B-cells at concentrations higher than 0.1 mg/L Ca^2+^ (Figure 5E).

**Figure 6:**
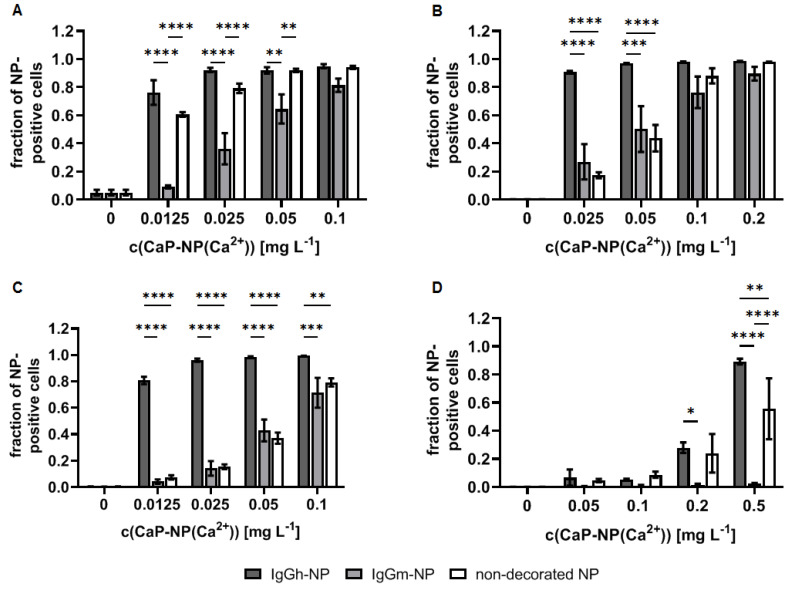
IgG of xenogenic origin exhibit high uptake rates compared to allogenic origin or non-decorated CaP/PEI-Cy5/SiO_2_ nanoparticles in cellular players of inflammation. Uptake in murine B-cells ((**A**) MOPC-315), endothelial cells ((**B**) SVEC-10), monocytes ((**C**) J774A.1), and T-cells ((**D**) TK-1) after 24 h of incubation. Cells were analyzed by flow cytometry (fraction of Cy5-positive cells after nanoparticle accumulation). Nanoparticles were decorated with Armenian hamster (IgGh) or murine IgG (IgGm) antibodies without any target specificity to cells under investigation. For detailed specification of nanoparticle formulations used, see Table 1 and Table 2. Statistical differences between indicated groups with * *p* < 0.05, ** *p* < 0.01, *** *p* < 0.001, **** *p* < 0.0001.

The comparison of the nanoparticle behavior in cellular players of inflammation showed that the cellular accumulation can be increased when they are decorated with xenogenic IgG vs. allogenic ones (Figure 6). Namely, there is a very strong accumulation in B-cells, endothelial cells, and monocytes, but a comparatively lower one in T-cells. The effect is particularly prominent at low nanoparticle concentrations (0.01 mg/mL Ca^2+^) and weaker at higher nanoparticle concentrations (0.1 mg/mL Ca^2+^). In contrast, the uptake is suppressed in the presence of allogenic IgG, particularly in B-cells and in T-cells, but to a lesser extent in endothelial cells and monocytes.

## 4. Discussion

Independently from the type of nanoparticle surface decoration (with either RGD peptide or IgG antibodies), the calcium phosphate nanoparticles had similar physicochemical properties. Additionally, our data showed that (1) our RGD-decorated nanoparticles are efficient in down-regulating p65 NF-κB protein expression in endothelial cells as a result of increased specific binding uptake of nanoparticles in those cells and (2) that nanoparticles decorated with IgG with specificity for CD69 are efficient in down-regulating p65 NF-κB protein expression in T-cells but not in B-cells, whereas the nanoparticle uptake in those cells was mediated, at least in parts, by IgG-based (but non-CD69-specific) nanoparticle binding. Low-dose nanoparticle decoration with xenogenic IgG stimulated their uptake in leucocytes and endothelial cells. The knowledge of mentioned biological interactions of the nature and surface functionalities of therapeutic nanoparticles should be helpful when addressing immune cells with NF-κB-specific siRNAs in the future.

The combination of the targeting moiety RGD-peptide with the p65 functional siRNA led to a down-regulation of p65 NF-κB in endothelial cells. Interestingly, the presence of RGD in unloaded nanoparticles (or in nanoparticles with encapsulated non-functional siRNA) was rather stimulating in terms of p65 expression in those cells. Although the underlying reasons are not yet clear, we tentatively postulate that the binding of the nanoparticles to RGD per se exerts a stimulatory effect on p65 NF-κB expression, and that p65 plays a particular role in this process. In this view, it has been demonstrated in a number of studies that integrin binding rapidly enhances the NF-κB activity (e.g., [9,22]). The nanoparticles are internalized into the endothelial cells via membrane invagination [20,21]. The internalized calcium phosphate nanoparticles are expected to be degraded in endo-lysosomes and the siRNA cargo released into the cytoplasm [12], where it can exert its gene-silencing effect.

The RGD peptide-decorated nanoparticles carrying p65 siRNA are effective in attenuating inflammation and inflammation-associated diseases, where p65 NF-κB is involved in cell growth, mediator secretion, and many other processes [9], especially if there is a favorable balance between the RGD-mediated cellular uptake and the intracellular availability of anti-p65 siRNA. Beyond the few studies showing the impact of injecting non-encapsulated RGD-p65-siRNA nanoparticle conjugates in mice with rheumatoid arthritis (1 mg/mL i.v. tail vein injection [23]), the particular advantage of using multi-shell calcium phosphate nanoparticles is the fact that the siRNA can be protected from degradation in the blood. We postulate that the therapeutic concentration of RGD-decorated calcium phosphate siRNA nanoparticles should be determined thoroughly also from the viewpoint that very high silica nanoparticle concentrations (25, 50, 100, and 200 µg/mL) were reported to activate NF-κB pathways by induction of oxidative stress [24].

Our IgG-decorated nanoparticles carrying siRNA were efficient in down-regulating p65 NF-κB in T-cells, particularly when carrying antibodies against CD69 and non-functional siRNA. This result could be attributed to the multi-faceted role of NF-κB in inflamed T-cells, involving not only the canonical, i.e., p65-mediated pathway, but also the non-canonical one with additional NF-κB players, such as RANK (receptor activator of NF-κB), p100 (a member of the NF-κB protein family), NIK (NF-κB-binding kinase), or JNK (c-Jun NH_2_-terminal kinase) [25,26,27,28]. In T-cells, the nanoparticle decoration with IgG distinctly favored its binding and accumulation, compared to the non-decorated nanoparticles (plain, or SH-terminated). Nevertheless, the nanoparticle accumulation in those cells was lower compared to the other immune cells investigated in this study. Moreover, the IgG-mediated nanoparticle binding to T-cells was, at least in parts, specific for CD69. From competition experiments and derived from the fact that the receptor CD69 is internalized upon binding and degraded afterwards [17], we deduce that CD69-targeting promotes nanoparticle internalization into T-cells. Further nanoparticle internalization into T-cells could have happened with FcγR, where the Fc-portion of the IgG of the nanoparticle surface acted as ligand, since competition experiments have shown a weak but discernible specificity for IgG-mediated accumulation. Moreover, it is well known that T-cells express FcγR only during a narrow window following cellular activation [29]. The internalization of IgG-decorated nanoparticles into T-cells occurred non-specifically as our competition experiments showed. Potential targets are non-specific membrane invaginations after interactions between the IgG molecules and the glycocalyx of cells. Given the curvature and size of the nanoparticles, we postulate a receptor-mediated endocytosis followed by degradation in endolysosomes, and the delivery of free siRNA into the cytoplasm [12].

In B-cells, the down-regulation of p65 NF-κB was not visible despite a comparatively strong non-IgG-based nanoparticle uptake by these cells. In this case, the nanoparticle accumulation was neither CD69- nor FcγR-specific, as our competition experiments showed. The reasons for such a relatively high non-specific B-cell accumulation of IgG-decorated nanoparticles are unknown, and they may well be associated with unspecific endocytosis (see above) or MHCII recognition processes. The rather up-regulation of p65 NF-κB protein expression in B-cells could be associated with the fact that non-specific nanoparticle bindings per se activated NF-κB pathways. For example, there is a known cross-talk between the NF-κB and Fc-receptor signaling [30], and furthermore, a cross-talk between CD69-related JAK/STAT and the NF-κB signaling pathway [17,31]. In this view, it is conceivable that the specific binding of CD69 receptors on B-cells via anti-CD69 IgG-molecules on NPs may have well triggered NF-κB expression. Furthermore, it is generally known that IgG molecules contain Fc-regions and that B-cells express Fc- receptors opposed to T-cells [32]. Besides the mentioned specific CD69 receptor-mediated NP-binding, an unspecific binding could have occurred between the Fc-regions of the IgG-based NP surface decoration and Fc surface receptors of B-cells. Such interactions could have activated NF-κB signaling, and consequently, led to p65-NF-κB up-regulation. Since the process of NP uptake occurs earlier in time than the NP cargo release into endolysosomes and cytoplasma [33], the intracellularly delivered anti-p65 siRNA was not able to reverse the preceded p65-NF-κB up-regulation upon NP-binding to B-cells. This view is in agreement with our observation that the Lipofectamine^TM^-mediated transfection of free p65 functional siRNA into B-cells was effective in reducing their p65 NF-κB protein expression (absence of NP-encapsulated siRNA) as opposed to the IgGsurface decorated NPs. This means that upon administration in vivo, IgG-decorated nanoparticles will be effective in down-regulating p65 NF-κB protein expression in endothelial cells and T-cells but not in B-cells.

In the present study, we generally refer to the total amount of p65 NF-κB protein, and not directly to the activated p65 NF-κB protein, which is known to be translocated to the cell nucleus. This means that the siRNA-mediated inhibition of the p65 NF-κB protein will not necessarily correlate with the functional inhibition of NF-κB activity. To verify such correlations, further in vitro and in vivo animal experiments should be conducted in the future. Another point to be addressed in the long run is the number of nanoparticles internalized by inflamed cells of the blood compartment and their therapeutic efficacy in the in vivo situation.

The fact that nanoparticles decorated with xenogenic IgG were better accumulated by all investigated cell types may potentially open up a strategy to regulate the intracellular availability of siRNA in the cytoplasm of specific immune cells. We expect that nanoparticle accumulation occurred mainly via non-specific processes, such as MHCII recognition in the investigated cells. Nevertheless, special attention should be paid to the fact that some unwanted activation of NF-κB-mediated pro-inflammatory pathways may occur in association with its recognition as foreign antigen [34]. A potential strategy may be accomplished by decorating calcium phosphate nanoparticles with very small amounts of xenogenic IgGs or technologically-modified IgGs, which could act as “subliminal baits” for immune cells, particularly for those which have a low intrinsic propensity to accumulate calcium phosphate nanoparticles, such as T-cells.

## 5. Conclusions

In summary, our data reveal that the composition of the calcium phosphate nanoparticles and the presence of a peptide- or antibody-based targeting moiety on their surface have an impact on the p65 NF-κB protein expression in different immune cells, and such effects can either stimulate or even weaken the functionality of the encapsulated functional siRNA against p65. Finally, a smart nanoparticle decoration with xenogenic IgG may stimulate their uptake in certain immune cells, which are otherwise difficult to address. Such interrelations have seldom been taken into account so far. It is vitally important to understand said interactions; this will allow us optimizing the functionalities of therapeutic nanoparticle-based biomedicines more specifically when addressing immune cells with anti-NF-κB siRNAs.

## Figures and Tables

**Table 1:** Characterization data of cRGDfK-decorated calcium phosphate nanoparticles used for in vitro cell targeting and gene silencing of NF-κB p65 in endothelial cells. Nanoparticles without ligand decoration and siRNA loading (CaP/PEI-Cy5/SiO_2_/SH/H_2_O, CaP/PEI-Cy5/SiO_2_/SH and CaP/PEI-Cy5/SiO_2_) were used as reference nanoparticles for all types of ligand-decorated nanoparticles. CaP—calcium phosphate, Cy5—cyanine 5, DLS—dynamic light scattering, EU—endotoxin unit, PDI—polydispersity index, PEI—polyethyleneimine, SEM—scanning electron microscopy, siRNA—small interfering ribonucleic acid, siRNAf—functional siRNA, siRNAs—scrambled siRNA. * Calcium concentration below the AAS detection limit. ** Estimated data based on the result of calcium concentration below the AAS detection limit.

Short Denomination	RGD-F-NP	RGD-S-NP	RGD-NP	SH-NP	NP^(+)^
Summarized Description	CaP/PEI-Cy5/ siRNAf/SiO_2_/ S-cRGDfK	CaP/PEI-Cy5/ siRNAs/SiO_2_/ S-cRGDfK	CaP/PEI-Cy5/ SiO_2_/S-cRGDfK	CaP/ PEI-Cy5/ SiO_2_/SH	CaP/ PEI-Cy5/ SiO_2_
Size by SEM (core diameter)/nm	59 ± 8	67 ± 7	49 ± 9	39 ± 4	57 ± 8
Size by DLS (hydrodynamic diameter)/nm	230 ± 4	325 ± 25	212 ± 3	211 ± 3	135 ± 3
PDI	0.24 ± 0.04	0.35 ± 0.04	0.19 ± 0.03	0.23 ± 0.01	0.19 ± 0.07
Zeta potential/mV	41 ± 1	39 ± 1	31 ± 2	29 ± 1	28 ± 2
[Ca^2+^]/µg mL^−1^	<7 *	<7 *	19	41	36
[CaP]/µg mL^−1^	<18 **	<18 **	48	103	91
[siRNA]/µg mL^−1^	33	20	-	-	-
siRNA molecules per nanoparticle	>28,700 **	>25,500 **	-	-	-
[peptides] µg mL^−1^	95	100	92	-	-
Peptide molecules per nanoparticle	>1.8 ∙ 10^6^ **	>2.8 ∙ 10^6^ **	3.7 ∙ 10^5^	-	-
[PEI]/µg mL^−1^	146	174	102	126	128
[Endotoxins]/EU mL^−1^	0.02	0.02	0.03	0.04	0.05
Nanoparticles/mL^−1^	<5.13 ∙ 10^10^ **	<3.62 ∙ 10^10^ **	2.52 ∙ 10^11^	1.03 ∙ 10^12^	2.84 ∙ 10^11^

^(+)^ non-decorated.

**Table 2:** Characterization data of antibody-decorated calcium phosphate nanoparticles used for in vitro cell targeting and gene silencing of p65 NF-κB in lymphocytes. For characterization of reference nanoparticles and abbreviations, see Table 1.

Short Denomination	CD69-F-NP	CD69-S-NP	CD69-NP	IgG-F-NP	IgG-S-NP	IgGh-NP	IgGm-NP
Summarized Description	CaP/PEI-Cy5/ siRNAf/SiO_2_/ S-IgGh- anti-CD69	CaP/PEI-Cy5/ siRNAs/SiO_2_/ S-IgGh- anti-CD69	CaP/ PEI-Cy5/ SiO_2_/ S-IgGh- anti-CD69	CaP/ PEI-Cy5/ siRNAf/ SiO_2_/ S-non- spec-IgGh	CaP/ PEI-Cy5/ siRNAs/ SiO_2_/ S-non-spec- IgGh	CaP/ PEI-Cy5/ SiO_2_/ S-non- spec-IgGh	CaP/ PEI-Cy5/ SiO_2_/ S-non-spec- IgGm
Size by SEM (core diameter)/nm	38 ± 5	50 ± 6	36 ± 3	36 ± 4	62 ± 9	44 ± 7	36 ± 3
Size by DLS (hydrodynamic diameter)/nm	189 ± 7	205 ± 7	212 ± 4	224 ± 11	218 ± 1	206 ± 6	145 ± 4
PDI	0.26 ± 0.06	0.21 ± 0.04	0.28 ± 0.03	0.33 ± 0.06	0.28 ± 0.02	0.27 ± 0.04	0.25 ± 0.02
Zeta potential/mV	37 ± 4	29 ± 1	31 ± 2	32 ± 1	27 ± 1	43 ± 1	34 ± 4
[Ca^2+^]/µg mL^−^^1^	13	34	13	20	16	<7 *	13
[CaP]/µg mL^−1^	33	86	33	51	41	<18 **	33
[siRNA]/µg mL^−1^	33	29	-	29	29	-	-
siRNA molecules per nanoparticle	4135	3165	-	2008	12,824	-	-
[Antibody]/µg mL^−1^	137	267	120	160	243	121	153
Antibody molecules per nanoparticle	1487	2580	1088	1006	9476	>3876 **	1424
[PEI]/µg mL^−1^	111	141	127	176	158	146	138
[Endotoxins]/EU mL^−1^	0.01	0.02	0.04	0.01	0.07	0.02	0.09
Nanoparticles/mL^−1^	3.62 ∙ 10^11^	4.17 ∙ 10^11^	4.44 ∙ 10^11^	6.37 ∙ 10^11^	1.03 ∙ 10^11^	<1.26 ∙ 10^11^ **	4.32 ∙ 10^11^

IgGh and IgGm: IgG from hamster and mouse, respectively.

## Data Availability

Not applicable.

## Supplementary Materials

The following supporting information can be downloaded at: https://www.mdpi.com/article/10.3390/biomedicines10071571/s1: Supplementary Figure S1: Demonstration of the calcium loss from calcium phosphate nanoparticles during synthesis, Supplementary Figure S2: Physicochemical properties of cRGDfK-decorated calcium phosphate nanoparticles and of control samples, Supplementary Figure S3: Physicochemical features of the antibody-decorated calcium phosphate nanoparticles.
