# Supplementary material for: The Peptide/Antibody-Based Surface Decoration of Calcium Phosphate Nanoparticles Carrying siRNA Influences the p65 NF-κB Protein Expression in Inflamed Cells In Vitro
Source: Biomedicines. 2022 Jul 1;10(7):1571. doi: 10.3390/biomedicines10071571 (PMC9313450; doi:10.3390/biomedicines10071571)
Supplement: Supplementary file 1 [file biomedicines-10-01571-s001.zip › biomedicines-1787188-supplementary.pdf]

biomedicines

MDPI

Supplementary information

The peptide/antibody-based surface decoration of calcium phosphate nanoparticles carrying siRNA influences the p65 NF-κB protein expression in inflamed cells *in vitro*

Elena K. Müller [1+], Nataniel Białas[2+], Matthias Epple[2*], and Ingrid Hilger[1*]

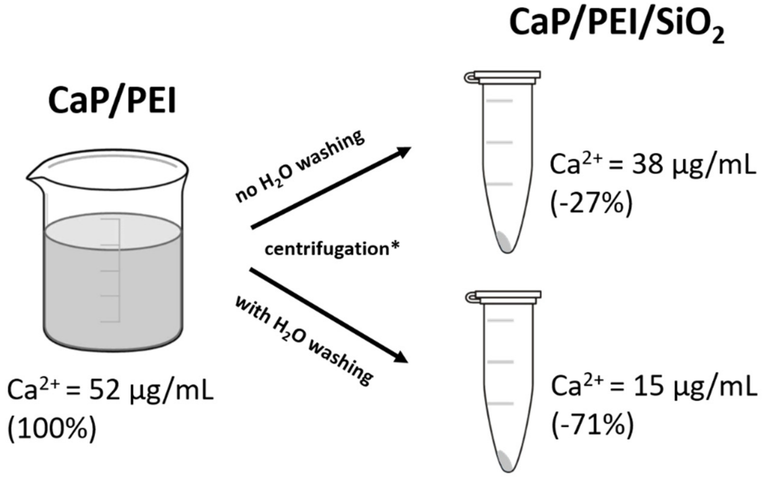

Supplementary Figure S1. Demonstration of the calcium loss from calcium phosphate nanoparticles during synthesis. Non-functionalized (CaP/PEI) and silica-functionalized (CaP/PEI/SiO2) nanoparticles were investigated. Nanoparticle pellets, obtained after centrifugation, were analyzed by AAS for the calcium concentration. CaP – calcium phosphate, PEI – polyethyleneimine. *centrifugation conditions: 1,537 x g, 30 min, RT. Beaker and reaction tube images were adapted from: www.paintingvalley.com and www.clker.com, respectively.

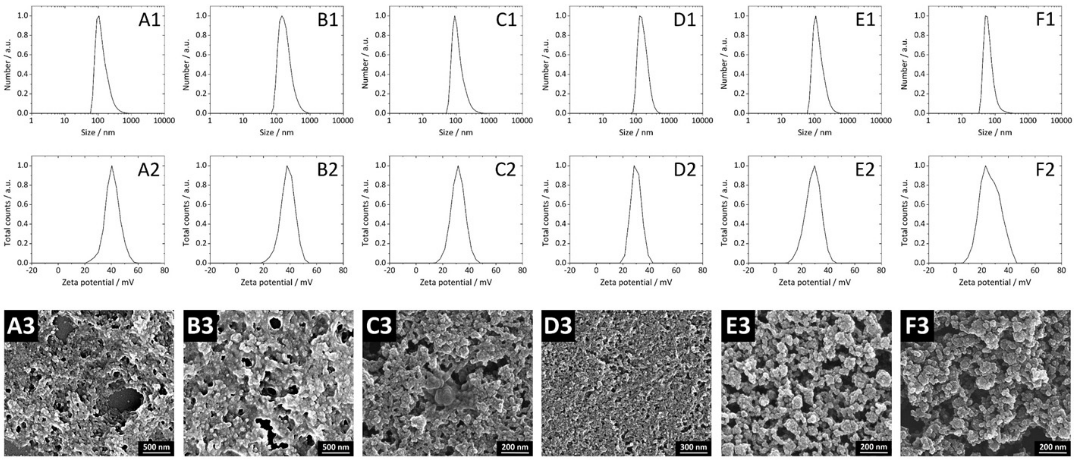

Supplementary Figure S2. Physicochemical properties of cRGDfK-decorated calcium phosphate nanoparticles and of control samples. Normalized particle size distributions (**A1**-**F1**) and the corresponding zeta potentials (A2-F2) by DLS, and the SEM micrographs of the nanoparticles (**A3**-**F3**). The nanoparticle formulations are presented as follows: RGD-F-NP (CaP/PEI-Cy5/siRNAf/SiO_2/S-cRGDfK) (**A**), RGD-S-NP (CaP/PEI-Cy5/siRNAs/SiO_2/S-cRGDfK) (**B**), RGD-NP (CaP/PEI-Cy5/SiO_2/S-cRGDfK) (**C**), SH-NP (CaP/PEI-Cy5/SiO_2/SH/H_2O) (**D**), SH-NP (CaP/PEI-Cy5/SiO_2/SH) (**E**), and NP (non-decorated, CaP/PEI-Cy5/SiO_2) (**F**).

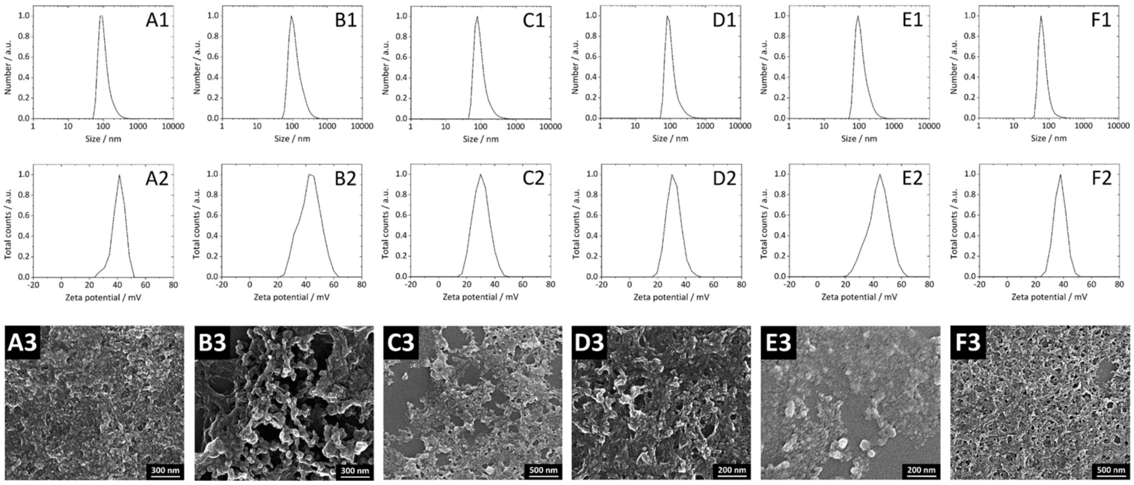

Supplementary Figure S3. Physicochemical features of the antibody-decorated calcium phosphate nanoparticles. Normalized particle size distributions (**A1-F1**) and the corresponding zeta potentials (**A2-F2**) by DLS, and the SEM micrographs of the nanoparticles (**A3-F3**). The nanoparticle formulations are presented as follows: CD69-F-NP (CaP/PEI-Cy5/siRNAf/SiO_2/S-IgG-anti-CD69) (**A**), CD69-S-NP (CaP/PEI-Cy5/siRNAs/SiO_2/S-IgG-anti-CD69) (**B**), CD69-NP (CaP/PEI-Cy5/SiO_2/S-IgG-anti-CD69) (**C**), IgGh-F-NP (CaP/PEI-Cy5/siRNAf/SiO_2/S-IgG-anti-CD69-CTRL) (**D**), IgGh-S-NP (CaP/PEI-Cy5/SiO_2/S-IgG-anti-CD69-CTRL) (**E**), and IgGm-NP (CaP/PEI-Cy5/SiO_2/S-IgG-κ-IC) (**F**). DLS and SEM results for CaP/PEI-Cy5/siRNAs/SiO_2/S-IgG-anti-CD69-CTRL nanoparticles are presented in the main text of the manuscript. DLS and SEM results for the control nanoparticles without siRNA loading and ligand decoration are presented in the Supplementary Figure S2.
